# Smartphone apps for depression and anxiety: a systematic review and meta-analysis of techniques to increase engagement

**DOI:** 10.1038/s41746-021-00386-8

**Published:** 2021-02-11

**Authors:** Ashley Wu, Matthew A. Scult, Emily D. Barnes, Jessica A. Betancourt, Avital Falk, Faith M. Gunning

**Affiliations:** 1 MD Program, Weill Cornell Medicine, New York, NY USA; 2 Department of Psychiatry, Weill Cornell Medicine, New York, NY USA

**Keywords:** Anxiety, Depression, Psychology

## Abstract

Meta-analyses have shown that digital mental health apps can be efficacious in reducing symptoms of depression and anxiety. However, real-world usage of apps is typically not sustained over time, and no studies systematically examine which features increase sustained engagement with apps or the relationship between engagement features and clinical efficacy. We conducted a systematic search of the literature to identify empirical studies that (1) investigate standalone apps for depression and/or anxiety in symptomatic participants and (2) report at least one measure of engagement. Features intended to increase engagement were categorized using the persuasive system design (PSD) framework and principles of behavioral economics. Twenty-five studies with 4159 participants were included in the analysis. PSD features were commonly used, whereas behavioral economics techniques were not. Smartphone apps were efficacious in treating symptoms of anxiety and depression in randomized controlled trials, with overall small-to-medium effects (*g* = 0.2888, SE = 0.0999, z(15) = 2.89, *p* = 0.0119, Q(df = 14) = 41.93, *p* < 0.0001, *I*^2^ = 66.6%), and apps that employed a greater number of engagement features as compared to the control condition had larger effect sizes (β = 0.0450, SE = 0.0164, t(15) = 2.7344, *p* = 0.0161). We observed an unexpected negative association between PSD features and engagement, as measured by completion rate (β = −0.0293, SE = 0.0121, t(17) = 02.4142, *p* = 0.0281). Overall, PSD features show promise for augmenting app efficacy, though engagement, as reflected in study completion, may not be the primary factor driving this association. The results suggest that expanding the use of PSD features in mental health apps may increase clinical benefits and that other techniques, such as those informed by behavioral economics, are employed infrequently.

## Introduction

Digital mental health interventions are proliferating rapidly, garnering heightened interest from the public as well as the scientific community. Well over 10,000 mental health apps are now available for download^1^, and multiple meta-analyses demonstrate that apps are modestly efficacious in treating symptoms of depression and anxiety^2–5^. However, an analysis of real-world usage of mental health apps show that user engagement over time is low, with a median 15-day retention of only 3.9%^6^. Apps with features that increase engagement have generally been associated with greater reductions in depression and anxiety symptoms than apps that lack engagement features^5^. No study systematically examines which features increase engagement with apps or the relationship between the number of engagement features and clinical efficacy.

Persuasive system design (PSD) is a framework that identifies four mechanisms through which app features increase user engagement: facilitating the primary purpose of the app, promoting user-app interactions, leveraging social relationships, and increasing app credibility^7^ (Supplementary Table 1). PSD is rooted in research on human-computer interaction and computer-mediated communication and is in part adapted from concepts of persuasive technology^7,8^. These categories allow for comprehensive and objective consideration of the characteristics of technologies. Specific PSD features predict adherence among internet-based health and lifestyle interventions, which suggests that incorporating PSD features may improve adherence in health apps as well ^7^.

Behavioral economics is focused on understanding ways that human decision-making differs from what would be expected in traditional economic models of “rational” actors^9^. A comprehensive list of behavioral economics techniques has not been delineated, but common findings include that people are motivated by avoiding losses^10^, “starting fresh,”^11^ committing to action^12^, and by lotteries^13^ (Supplementary Table 2). These techniques from behavioral economics can be applied to “gamification,” the use of game-like experiences in non-game services, to further improve user engagement^14–17^. Gamification features, including points, badges, levels, and avatars, are used to increase engagement and may enhance apps’ intended effects^16^. In one head-to-head comparison of a gamified vs non-gamified versions of a smartphone app for anxiety, the gamified app was found to elicit significantly greater engagement, demonstrating the potential of behavioral economics for improving engagement with mental health apps^18^. PSD, behavioral economics, and gamification often overlap and identify a wide variety of techniques that increase engagement with smartphone apps.

Though smartphone apps show potential for treating depression and anxiety, a barrier to their real-world efficacy may be a lack of sustained user engagement^19^. Understanding the extent to which the engagement features of PSD techniques, behavioral economics, and/or gamification translate into improvements in engagement and mood outcomes may inform the development and deployment of more efficacious apps for anxiety and depression. We therefore conducted a systematic review of the literature and meta-analysis on smartphone apps for depression and anxiety with the aim of evaluating the impact of persuasive design and behavioral economics techniques on both engagement and clinical outcomes.

## Results

### Included studies

**Fig. 1:**
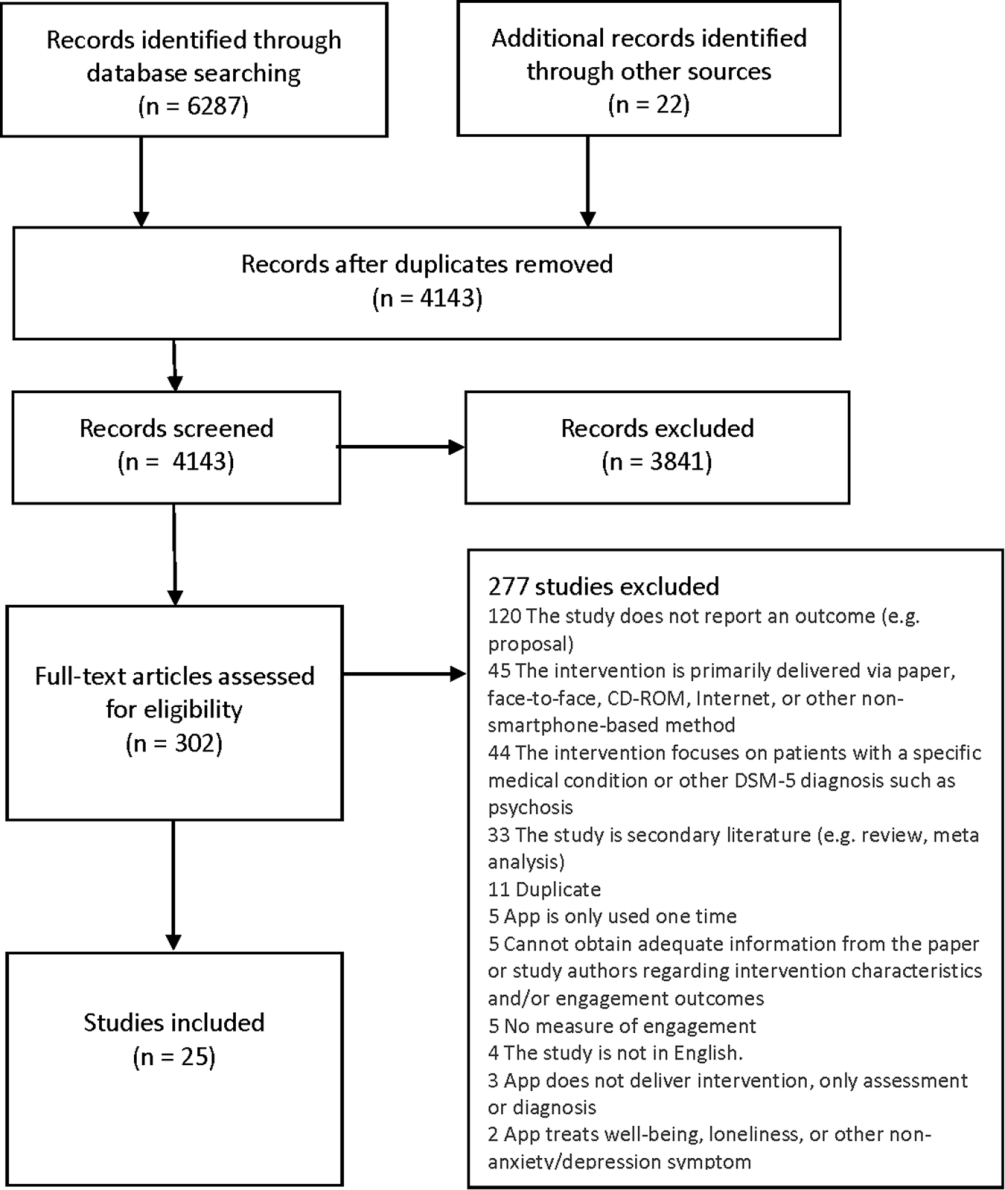
PRISMA flowchart. Flow diagram of article selection process.

The full systematic search retrieved a total of 6287 results. Following the removal of duplicate articles and the later addition of articles cited by included studies, 4143 articles were screened by the title and abstract for relevance. After title and abstract screening, 302 articles were identified as potentially eligible and were screened in full. Full-text screening excluded 277 articles for reasons specified in Fig. 1, which details the full PRISMA search and screening process.

After full text screening, 25 independent studies were found to be eligible for inclusion in the systematic review (Table 1)^20–44^. Ten studies were non-randomized trials, while 15 were randomized controlled trials. In addition, 3 of the trials were unpublished, including one pre-print and two dissertations. Across all studies, there were 4159 participants total, with 2905 participants using 29 unique smartphone apps. The participants ranged from 13 to 76 years old, with an average age of 32.9. The majority of participants were female (66.6%). The majority of studies required participants to report significant symptoms of major depressive disorder (19) or generalized anxiety disorder (5), and symptoms were most commonly identified by scoring above a predetermined threshold on a validated self-assessment questionnaire.

**Table 1:** Study characteristics.

**Type of study (*N*** **=** **25)**	
RCT	15 articles
Pilot/quasi experimental	10 articles
Gray literature (*N* = 3)	
Dissertation	2 articles
Pre-Print	1 article
**Apps (*N*** **=** **29)**	
Participant demographics (*N* = 4159)	
Average age (Min–Max) (years)	32.9 (13–76)
Female (%)	66.6
**Diagnosis of study participants**	
Major depressive disorder	19 studies
Generalized anxiety disorder	5 studies
Social anxiety disorder	3 studies
Dysthymia	1 study
Other depressive disorder	1 study
**Method of symptom evaluation**	
Self-identification	2 studies
Validated self-assessment measure	19 studies
Clinician-administered measure or medical record	8 studies
**Study length (Weeks)**	
Average Length of Intervention (Min–Max)	5.82 (2–12)

Control conditions included waitlist, treatment as usual, non-app control intervention, or placebo app control conditions (Supplementary Table 3). Average intervention duration was 5.8 weeks (standard deviation 2.8 weeks), with a range of 2–12 weeks. Most studies accounted by drop out by conducting an intention to treat analysis or by removing missing cases (Supplementary Table 4).

### Quality assessment

According to the Joanna Briggs Institute critical appraisal checklist for quasi-experimental studies^45^, among the 10 quasi-experimental studies, 2 were rated to be high quality, 6 were medium quality, and 2 were low quality. According to the Cochrane Collaboration Revised Risk of Bias tool^46^, among the 15 randomized controlled trials, 0 were judged to have an overall low risk of bias, 8 were judged to raise some concerns for bias, and 7 were judged to be at high risk of bias.

### App content

Across all studies, 29 unique smartphone apps were used, including experimental and control apps. The apps offered a variety of content (Supplementary Table 5), the most common being behavioral techniques (76%), cognitive techniques (72%), and psychoeducation (69%). Some apps provided means to track behavior (45%), mood (38%), or thoughts (21%). Relaxation techniques and mindfulness techniques were each used in 31% of apps. Fewer apps tracked physiological parameters such as sleep or exercise (10%) or tracked clinical symptoms of anxiety or depression (7%).

### Safety and privacy

Safety features such as assessing for suicidality and providing resources for suicidality were reported in a minority of apps (14% and 17%, respectively). For 20% of apps, a privacy policy was available as specified in the study. Additional privacy and security features such as encryption and password protection were reported for 27% of apps (Supplementary Table 6).

### Accessibility

Of the 29 apps assessed, 41% were available to the public through the Apple App Store, Google Play, or both (Supplementary Table 7). The remaining apps were accessible to research participants only, or their accessibility was not specified. The majority of apps were free or had free versions available.

### Persuasive system design

Overall, apps used an average of 6.5 out of the 28 total features of PSD and 2.5 of the 4 total categories of PSD (Tables 2 and 3). Primary task support was most commonly used, with an average of 3.30 (SD 1.26) out of 7 total features. Social support was least commonly used, with an average of 0.37 (SD 0.93) out of 7 total features.

**Table 2:** Components of persuasive design.

App	Primary task support	Dialogue support	Social support	Credibility support
Aptivate^27^	X	X		
BA Intervention^33^	X	X		X
Be Good to Yourself^31^	X	X		X
Boost Me^40^	X	X		
Calm^37^	X	X		
CBMA^28^	X	X		
CBMA Control^28^	X	X		
El Buen Consejo Movil^24^	X	X	X	X
EVO^20,38^	X	X		X
FOCUS^22^	X	X		X
Get Happy Program^43^	X	X		X
Health Tips^20,38^	X	X		
iCBT^41^	X			
iCouch CBT^27^	X			
Intellicare^25,36^	X	X		X
Mindfulness App^33^	X			X
Mobile Sensing and Support^42^	X	X		
Moodivate^26^	X	X		
MoodKit^26^	X	X		
MoodMission^21^	X	X		
MT-Phoenix^32^	X	X		
Pacifica^23,35^	X	X	X	X
Plus Connect^44^	X	X	X	X
PUSH-D^34^	X	X		
Superbetter CBT^39^	X	X	X	X
Superbetter General^39^	X	X	X	X
Thought Challenger^41^	X			
Todac Todac^29^	X	X	X	
Wysa^30^	X	X		X

**Table 3:** Average persuasive system design features and categories.

Persuasive system design feature	Mean (SD)	Min	Max
Primary task support	3.30 (1.26)	1	6
Dialogue support	1.93 (1.20)	0	4
Social support	0.37 (0.93)	0	4
Credibility support	0.90 (1.24)	0	4
**Total features**	6.50 (2.60)	2	12
**Total categories**	2.50 (0.90)	1	4

Total features in each category = 7; total categories = 4.

Table 4 describes the number and percentage of apps that used each PSD feature or category. Of note, features of credibility support were most difficult to evaluate as most articles did not describe these features in detail, and 81.4% of ratings were marked as “unable to assess.” The most frequently used features (reduction − 86.7%, self-monitoring − 70.0%, personalization − 53.3%) all belonged to the primary task support category. The least frequently used features were not used in any of the apps (cooperation, competition, recognition, third-party endorsements, and verifiability) and belonged to the dialogue support category or credibility support category.

**Table 4:** Frequency of PSD features.

Feature	App with feature	% apps with feature
Reduction	26	86.7%
Tunneling	11	36.7%
Tailoring	12	40.0%
Personalization	16	53.3%
Self-monitoring	21	70.0%
Simulation	1	3.3%
Rehearsal	12	40.0%
**Total primary task support features across all apps**	**99**	
Praise	7	23.3%
Rewards	7	23.3%
Reminders	15	50.0%
Suggestion	16	53.3%
Similarity	5	16.7%
Liking	6	20.0%
Social role	2	6.7%
**Total dialogue support features across all apps**	**58**	
Social learning	3	10.0%
Social comparison	2	6.7%
Normative influence	1	3.3%
Social facilitation	5	16.7%
Cooperation	0	0%
Competition	0	0%
Recognition	0	0%
**Total social support features across all apps**	**11**	
Trustworthiness	7	23.3%
Expertise	7	23.3%
Surface credibility	10	33.3%
Real-world feel	1	3.3%
Authority	2	6.7%
Third party endorsements	0	0%
Verifiability	0	0%
**Total credibility support features across all apps**	**27**	

### Behavioral economics

Overall, apps used an average of 0.17 out of the 4 total behavioral economics features (Supplementary Table 8). The most commonly used was the pre-commitment pledge, which was used in 4 apps. One app used loss aversion, whereas none of the apps used the fresh start effect or lottery.

### Meta-analysis of effect of smartphone apps on depressive or anxiety symptoms

**Fig. 2:**
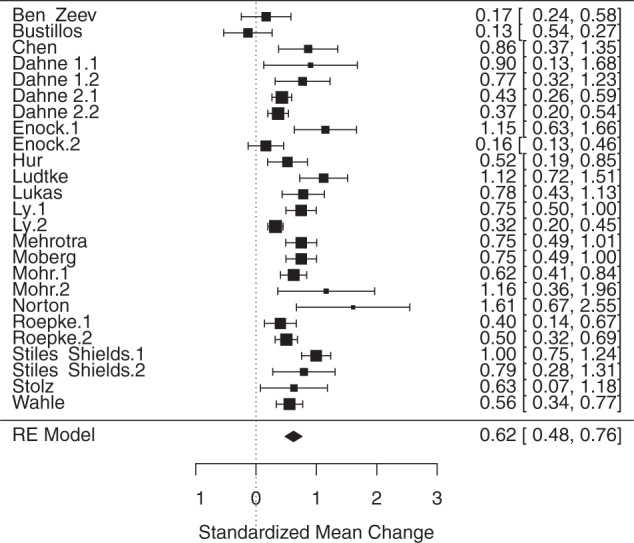
Random effects meta-analysis of app efficacy comparing pre-intervention to post-intervention change on an anxiety or depression symptoms. Forest plot of random effects meta-analysis of app efficacy comparing pre-intervention to post-intervention change on an anxiety or depression symptoms.

A random-effects meta-analysis of the effect of smartphone apps on depression and anxiety symptoms demonstrated a moderate positive effect size of smartphone apps for reducing pre versus post scores on depression and anxiety questionnaires (*g* = 0.6217, SE = 0.0669, t(25) = 9.2982, *p* < 0.001) (Fig. 2). Heterogeneity across studies was substantial (Q(df = 24) = 87.8894, *p* < 0.0001, *I*^2^ = 82.70%). Egger’s regression test indicated significant publication bias (*p* = 0.0135). A trim-and-fill analysis identified six missing studies (*g* = 0.5184, SE = 0.0803, z(31) = 6.4589, *p* < 0.001, Q(df = 30) = 125.7114, *p* < 0.0001, *I*^2^ = 87.57%).

**Fig. 3:**
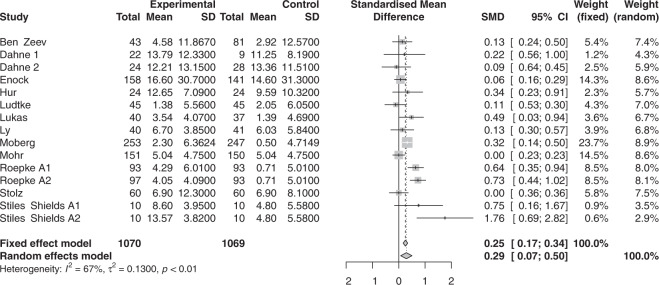
Random effects meta-analysis in RCTs of app efficacy comparing change in anxiety or depression symptoms in apps vs. control. Forest plot of random effects meta-analysis in RCTs of app efficacy comparing change in anxiety or depression symptoms in apps vs. control.

Figure 3 displays the pooled and individual effect sizes of smartphone apps on depression or anxiety when compared to control conditions in randomized controlled trials. A random-effects meta-analysis demonstrated a similar pattern as the pre/post meta-analysis of the full sample albeit with a smaller effect size (*g* = 0.2888, SE = 0.0999, z(15) = 2.89, *p* = 0.0119, Q(df = 14) = 41.93, *p* < 0.0001, *I*^2^ = 66.6%). Egger’s regression test indicated no publication bias (*p* = 0.47091). A trim-and-fill analysis identified one missing study (*g* = 0.2473, SE = 0.1254, t(16) = 1.97, *p* = 0.0672, Q(df = 15) = 49.72, *p* < 0.0001, *I*^2^ = 69.8%).

### Meta-regression of PSD features and efficacy

**Fig. 4:**
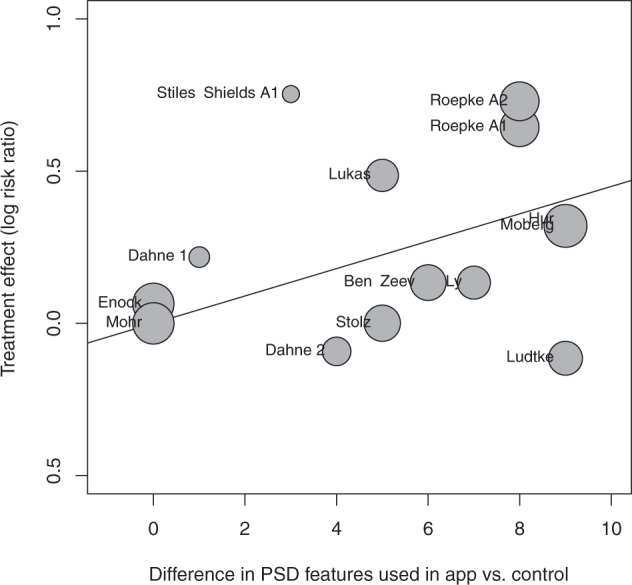
Mixed effects meta-regression analysis of difference in PSD features and efficacy for app vs control. Bubble plot of mixed effects meta-regression analysis of difference in PSD features and efficacy for app vs control.

Meta-regression analysis demonstrated that the magnitude of the effect size of improvement in mood in active relative to control conditions had a significant positive relationship with the difference in number of PSD features used in the app (β = 0.0450, SE = 0.0164, t(15) = 2.7344, *p* = 0.0161; Fig. 4), as well as with the difference in number of PSD categories (β = 0.1249, SE = 0.0362, t(15) = 3.4522, *p* = 0.0039).

### Meta-analysis of study completion rate

A random-effects meta-analysis of the study completion rate of app vs control conditions in randomized controlled trials revealed a significant effect, and heterogeneity was moderate to substantial (*g* = 0.8730, SE = 0.0761, z(17) = −1.79, *p* = 0.0931, Q(df = 16) = 41.33, *p* = 0.0005, *I*^2^ = 61.3%). Egger’s regression test indicated no publication bias (*p* = 0.2738). A trim-and-fill analysis identified no missing studies.

### Meta-regression of PSD features and study completion rate

**Fig. 5:**
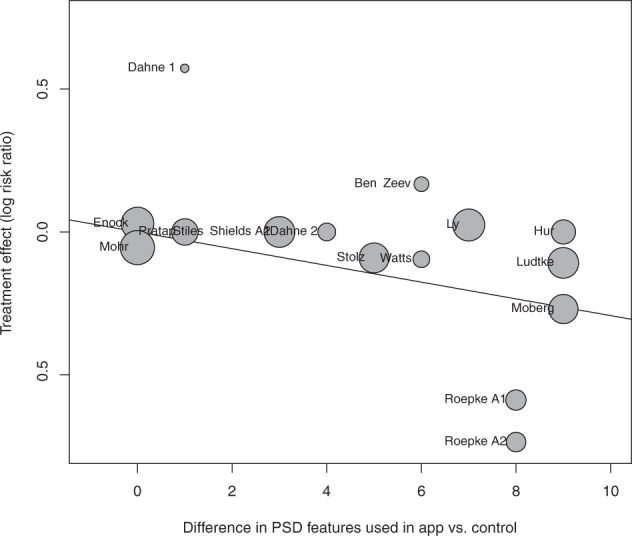
Mixed effects meta-regression analysis of difference in PSD features and study completion rate for app vs control. Bubble plot of mixed effects meta-regression analysis of difference in PSD features and study completion rate for app vs control.

The study completion rate in the intervention compared to control condition had a significant negative relationship with the difference in number of PSD features (β = −0.0293, SE = 0.0121, t(17) = 02.4142, *p* = 0.0281; Fig. 5), as well as with the difference in number of PSD categories (β = −0.0830, SE = 0.0292, t(17) = −2.8472, *p* = 0.0116).

Removal of outliers did not significantly impact any of the results (Supplementary Note 1).

### Exploratory analyses

No direct association was found between efficacy and completion rate (*g* = −0.2134, SE = 0.1442, t(15) = −1.4806, *p* = 0.1609). Efficacy also did not meaningfully change the association between completion rate and PSD features (β = −0.0649, SE = 0.1434, *p* = 0.6588, F(df1 = 2, df2 = 12) = 0.8320, *p* = 0.4588). Including a regressor for the type of control condition (waitlist vs. active control) also did not meaningfully change the association between PSD features and study completion rate (β = −0.1900, SE = 0.1254, t(17) = −1.5150, *p* = 0.1520). Including a regressor for initial symptom severity did not meaningfully change the association between PSD features and study completion rate (β = −0.0949, SE = 0.0820, t(16) = −1.1566, *p* = 0.2682).

## Discussion

The principal findings of this meta-analysis indicate that standalone smartphone apps for depression and anxiety are efficacious, with modest overall effects in randomized controlled trials (*g* = 0.2888). The efficacy findings are consistent with those shown in previous meta-analyses^2–5^. This meta-analysis is the first to demonstrate that apps that use a greater number of engagement features have larger clinical effects. Thus, the findings extend the extant literature by demonstrating the importance of engagement techniques to improve efficacy of app-based interventions for depression and anxiety. These findings can inform development and implementation of future apps.

A lack of engagement with mental health apps has been reported as a key limitation for realizing the potential of apps to broadly disseminate mental health treatments^47^. Apps that include guidance (i.e., from a therapist or coach) have generally been found to have larger effects than standalone interventions^5,19,48^. Given that standalone mental health apps require fewer resources than guided apps, identifying factors that might increase engagement and efficacy without therapist involvement could greatly improve dissemination efforts. “Engagement features” such as those elements of PSD have been found to increase engagement with mental health apps as measured by duration of use and completion of interventions^7,49^. Our review indicates that engagement features also are associated with increased clinical efficacy and therefore should be included in standalone mental health apps to increase their impact for broader dissemination.

The number of engagement features used in a given app had a significant negative association with study completion rate. The negative association between PSD features and engagement as measured by completion rate was contrary to our a priori hypothesis that PSD features would be associated with increased app completion and thereby increase efficacy. One possible interpretation of the negative association between PSD features and completion rates is that participants using apps with more PSD features may use the app more frequently, benefit more quickly, and lose motivation to continue using the app once their symptoms have improved compared to participants using less engaging apps; however, our exploratory analyses suggest that this is not the case. Additionally, we found that these results were not driven by the type of control condition or initial symptom severity. In subsequent studies, analyses of interim symptom change could help to better understand the association between PSD features and completion rates. Additionally, while completion rate was the most readily available measure of engagement, other engagement outcomes such as minutes spent using the app or self-reported user experience of the usefulness, usability, and satisfaction with the app^50^ might yield more meaningful information about PSD features and engagement.

We found that some PSD features were incorporated into all apps (e.g., primary task support), while others were less commonly used (e.g., social support), and behavioral economics features such as pre-commitment pledges, loss aversion, and lotteries, were rarely incorporated. The reason may be, in part, because of the historical roots of these literatures. PSD is well-established as an approach to development of new technologies and therefore may be more naturally incorporated into app development^7,8^. Behavioral economics has traditionally focused on economic decision-making^51^, then was applied to behavioral health^52^ but has only more recently been used in the context of mental health interventions^53^. The results of our meta-analysis suggest that increasing the number of engagement techniques may be beneficial, and so it is likely that designers may need to draw from a range of engagement approaches to maximize the impact of app-based interventions. Furthermore, elements such as social support, which span both the PSD and behavioral economics literatures, may be particularly promising^6^. Incorporating social support may also be the mostly likely way to engage patients without the use of therapists or coaches and, in itself, is a predictor of mental health outcomes ^54^.

There are several important limitations of the current review. The literature on mental health apps is heterogenous, and a number of studies included samples without psychiatric diagnoses and/or were used in combination with other interventions to varying degrees. Clearer standards for reporting on app content in research studies could help subsequent syntheses of the literature. In our review, all available information about app content, design elements, privacy and security features, and accessibility were extracted from descriptions and screenshots of the apps in the papers, appendices and supplementary materials. However, additional features may have been included in apps that were not described in these materials. Another limitation is the use of completion rate as the primary metric of engagement. While this approach has been used previously in mental health app engagement literature^19^ and is an accessible metric because it is reported in most studies, it may be a crude measure of engagement. The number of minutes spent using the app, number of logins, or number of modules completed (if applicable) may measure engagement more meaningfully^55^. Furthermore, the quality of engagement may be more likely to affect clinical outcomes as much as or even more than the duration of engagement. The issue of defining meaningful engagement has been gaining attention and will likely influence the next steps in measuring app engagement ^56^.

Overall, smartphone apps appear to be efficacious in decreasing anxiety and depression symptoms. The use of persuasive design features is associated with larger effect sizes and may be useful in increasing clinical efficacy. Relatively few mental health apps used non-PSD behavioral economics techniques, which have shown promise for increasing outcomes in app-based interventions for smoking cessation^57^, increasing physical activity for obese patients^58^, and adherence to diabetes medication^59^. Incorporating additional PSD and behavioral economics techniques that have been rarely used, but have shown promise (e.g., social support^7^) may further improve outcomes in mental health treatments.

## Methods

### Literature search

This systematic review was conducted in accordance with the Preferred Reporting Items for Systematic Reviews and Meta-Analyses (PRISMA) statement for transparent, comprehensive reporting of methodology and results^60^. The review adhered to a strict protocol submitted for registration with PROSPERO on April 1, 2020 (ID 171194) before the start of analyses. The protocol review was ongoing as of December 4, 2020 (CRD42020171194): https://www.crd.york.ac.uk/prospero/display_record.php?ID=CRD42020171194.

A systematic search was performed of the following databases: Ovid Medline (ALL − 1946 to present); Ovid Embase (1974 to present); The Cochrane Library (Wiley); the Cumulative Index to Nursing and Allied Health Literature (CINAHL; EBSCO interface); PsycINFO (EBSCO); PsycArticle (EBSCO); and Ovid Allied and Complementary Medicine (AMED). Since the focus of the study was on published research trials conducted on the apps, searches were not carried out directly in the Google Play or the Apple App Store. Our search terms included combinations, truncations, and synonyms of “depression,” “social anxiety,” or “anxiety,” in combination with “mobile application” or “smartphone.” The search terms were adapted for use with all chosen bibliographic databases. Additional records were retrieved by checking the bibliographies of studies that met inclusion criteria. No language, article type, or publication date restrictions were used. Searches were completed in March and April 2020.

### Eligibility criteria

Included articles were English-language empirical studies published in peer-reviewed publications, conference presentations, dissertations, and gray literature/unpublished work. The intervention was required to be delivered by smartphone app alone and to be delivered over time with the purpose of treating depression and/or anxiety (major depressive disorder, dysthymia, other depressive disorder, generalized anxiety disorder, and/or social anxiety disorder). Study participants could be any age but needed to identify as having depression and/or anxiety, either through clinical diagnosis or self-endorsement. To be included, studies had to report at least one measure of participant engagement (i.e., attrition, adherence, engagement, dropout).

Studies were excluded if: a) the intervention was delivered in part or entirely by a non-smartphone method; b) the study population was required to have a specific medical condition, other DSM-5 diagnosis, no diagnosis (healthy), or unspecified diagnosis, unless the study included a subgroup analysis of participants with depression and/or anxiety only; and c) adequate information regarding intervention characteristics and/or engagement outcomes could not be obtained from the paper or from contacting the study authors.

### Data collection and analysis

Two of three raters (E.B., J.B., or A.W.) independently completed each of the following steps, and conflicts were resolved by another rater (M.S.).

### Selection of articles

Titles and abstracts were evaluated against the inclusion and exclusion criteria. Studies that were deemed potentially relevant were next evaluated by their full text. Reasons for exclusion were recorded. When more than one publication of a study was found, the study with the most information was used for data analysis.

### Data extraction

Data extracted included study identifiers, study characteristics, intervention characteristics, techniques to increase engagement, engagement measures, efficacy measures, app content, app modality, accessory interventions, and usability features. Techniques to increase engagement are described further in Supplementary Information. Only data that were explicitly reported in the publications were extracted. App profiles in the Google Play or Apple App Store were not accessed as we could not verify that features identified in the app profile were available in the version of the app referenced in the paper.

1. Study Identifiers: the first author’s last name and the publication year.
2. Study Characteristics: the population, comparator (if applicable), outcome, and study design. Participant demographics such as age and gender.
3. Intervention Characteristics: the intervention or cohort name, number of trial arms, primary condition, duration of intervention, duration of follow-up, modules to be completed, automated or guided delivery, format of delivery, and outcome measures.
4. Techniques to Increase Engagement: components of PSD as outlined in Kelders et al.^7^ include: primary task support (reduction, tunneling, tailoring, personalization, self-monitoring, simulation, and rehearsal), dialogue support (praise, rewards, reminders, suggestion, similarity, liking, and social role), and credibility support (trustworthiness, expertise, surface credibility, real-world feel, authority, third-party endorsements, and verifiability). Non-PSD behavioral economics techniques included loss aversion, fresh start effect, pre-commitment pledges, and financial incentives including lotteries. Each component or technique had a predefined definition and guideline for being coded as present. Additional information can be found in Supplementary Tables 1 and 2.
5. Measures of Engagement: adherence to study protocol, completion rate, amount of time spent in app, modules completed, features or exercises accessed, duration of use of app, or other. All available measures of engagement were recorded for each study.
6. Measures of Efficacy: change in score on a validated scale for depression or anxiety.
7. App Content: app content was categorized based on categories identified in previously published systematic evaluations of publicly available mental health apps^6,61^. App content included psychoeducation, cognitive techniques, behavioral techniques, mindfulness, relaxation, mood expression, tracking of mood patterns, tracking of thought patterns, tracking of behavior, tracking of symptoms, and tracking of physiological parameters.
8. App Modality: information on app modality was recorded when available. App modalities included text, graphs, photos, illustrations, videos/films, and others.
9. Safety, Privacy, and Accessibility Features: integration and accessibility features such as cost, availability to the public, safety features, and privacy protection features. These features were adapted from a framework for evaluating mobile mental health apps^62^.

### Inter-rater agreement

At the title and abstract screening stage, inter-rater agreement was 93.8%. At the full-text screening stage, inter-rater agreement was 90.1%. At the engagement feature rating stage, inter-rater agreement was 82.6%. Disagreements were resolved by a third rater (M.S.) at the screening stages and by consensus at the feature rating stage.

### Assessment of risk of bias

Non-randomized experimental studies were evaluated based on the Joanna Briggs Institute critical appraisal checklist for quasi-experimental studies to assess quality^45^. Because the Joanna Briggs Institute checklist does not assign specific ratings (“high,” “medium,” or “low”) to studies based on the number of criteria met, we adapted a rating system used in previously published systematic evaluations of mental health apps^6,61^. The methodological quality of each study was rated as: high (7–9 criteria have been fulfilled), medium (4–6 criteria have been fulfilled), or low (0–3 criteria have been fulfilled).

The Cochrane Collaboration Revised Risk of Bias tool was applied to randomized controlled trials^46^. Each RCT was assessed against five bias domains in order to produce a summary risk of bias assessment score for each domain and overall (low risk, some concerns, or high risk of bias) ^46^.

### Data analysis

A meta-analysis was conducted to calculate a pooled effect size of treatment efficacy. To test pre- and post-test change scores on anxiety and depression questionnaires, we applied a random-effects model to account for the expected heterogeneity among the studies, given the variety of types of apps, duration of intervention, and other factors. If a study used multiple measures of anxiety and/or depression symptoms, then the instrument most frequently used among all studies was used to calculate efficacy so as to reduce heterogeneity. If an app was used in multiple independent studies, it was included multiple times. In supplementary analyses, we removed outliers, defined as studies with a 95% confidence interval of the effect size that did not overlap with the 95% confidence interval of the pooled effect size^63^. We compared analyses with and without outliers to determine whether the presence of outliers changed the observed results. Risk of publication bias was examined using Eggers’ regression, and Duval and Tweedie’s trim-and-fill analysis was used to re-calculate pooled dropout rates after statistically accounting for any studies that may have introduced publication bias^64^. The degree of statistical heterogeneity was quantified using *I*² values, with <25% representing low heterogeneity, 25–50% for low-to-moderate heterogeneity, 50–75% for moderate-to-substantial heterogeneity, and >75% for substantial heterogeneity^63^. τ^2^ was calculated using the Hartung–Knapp–Sidik–Jonkman (HKSJ) method, which is appropriate when there is a large degree of heterogeneity between studies.

A second meta-analysis was run to examine efficacy of apps compared to control conditions in randomized controlled trials. A random-effects model was again used given the expected heterogeneity among the studies. Efficacy was calculated as the pre/post change on a depression or anxiety scale. When a study had multiple control conditions, the active control condition was selected, as opposed to a waitlist or treatment-as-usual control. If an app was used in multiple studies, it was included multiple times. If a study used multiple apps, both apps were included if there was a non-app control condition. Otherwise, the intervention app was compared to the control app. To examine whether risk of bias score affected the efficacy, a subgroup analysis stratifying studies by risk of bias score was performed (Supplementary Note 1). Independent mixed-effects meta-regression models were run to examine how app efficacy was affected by the number of PSD features used in the app compared to the control condition, the number of PSD categories used in the app compared to the control condition, and the study completion rate of app users compared to the control condition. As the number of PSD features or categories denoted levels as opposed to continuous variables, the intercept was set to “false.”

A third meta-analysis examined the study completion rate of apps compared to control conditions in randomized controlled trials. Study completion rate was calculated as the proportion of enrolled and/or randomized study participants who remained in the study at the time the intervention ended. A random-effects model was again used given the expected heterogeneity among the studies. Procedures were otherwise the same as for the second meta-analysis and the meta-regression. In addition, in order to further explore the association between PSD features and completion rates, exploratory control analyses were conducted. An independent mixed-effects meta-regression model was run to examine how app efficacy affected study completion rate. Multiple meta-regressions were conducted to examine the relationship of various covariates to study completion rate: PSD features and efficacy, PSD features and type of control condition (waitlist vs. active control), and PSD features and initial symptom severity. Initial symptom severity was rated numerically based on the interpretation of the instrument used, with minimal or none rated as 0, mild as 1, moderate as 2, moderately severe as 3, and severe as 4. All analyses were conducted in R.

## Supplementary information

Supplementary Information

## Data Availability

The data used in this study are publicly available. The extracted data are available in the following github repository: https://github.com/ashlwu/SmartphoneAppsDepressionAnxiety. Of note, the data were analyzed in.csv format but have been converted to.xlsx format to allow the data to be password protected. The password is “Engage.”

## Code availability

Meta-analyses and meta-regressions were conducted using the Meta, Metafor, and Dmetar packages in R^65–67^. The code is publicly available in the following github repository: https://github.com/ashlwu/SmartphoneAppsDepressionAnxiety.
