# Supplementary material for: Smartphone apps for depression and anxiety: a systematic review and meta-analysis of techniques to increase engagement
Source: NPJ Digit Med. 2021 Feb 11;4:20. doi: 10.1038/s41746-021-00386-8 (PMC7878769; doi:10.1038/s41746-021-00386-8)
Supplement: Supplementary file 1 — Supplementary Information [file 41746_2021_386_MOESM1_ESM.pdf]

Supplementary Information

Supplementary Note 1: Analyses When Removing Outliers and Risk of Bias Subgroup Analyses

Meta-analysis of effect of smartphone apps on depressive or anxiety symptoms

Four outliers were identified for the pre/post meta-analysis, and upon exclusion of outliers, the effect size remained significant, and heterogeneity remained similar (g=0.6021, SE=-0.0775, t(21)=7.7650, p<0.0001, Q(df=20)=71.1345, p<0.0001, I^2=85.13%). Looking at just randomized controlled trials, one outlier was identified. When the outlier was removed, the effect size remained significant, and heterogeneity remained similar (g=0.2434, SE=0.1661, z(14)=3.17, p=0.0074, Q(df=13)=34.25, p=0.0011, I^2=62.0%). No subgroup differences in efficacy were detected when the studies were stratified by risk of bias score (Q(df=1)=1.45, p=0.2284).

Meta-regression PSD features and efficacy

When the one outlier was removed, the findings were similar for both PSD features (β=0.0420, SE=0.0111, t(14)=3.8010, p=0.0022) and PSD categories (β=0.1157, SE=0.0210, t(14)=5.4966, p=0.0001).

Meta-analysis of study completion rate

One outlier was identified. When the outlier was removed, the effect size remained non-significant, and heterogeneity decreased (g=0.9209, SE=0.0993, z(16)=-1.54, p=0.1441, Q(df=13)=21.52, p=0.1211, I^2=30.3%). No subgroup differences in efficacy were detected when the studies were stratified by risk of bias score (Q(df=1)=0.46, p=0.4991).

Meta-regression of PSD features and study completion rate

When the one outlier was removed, the findings were similar for PSD features (β=-0.0207, SE=0.0081, t(16)=-2.5424, p=0.0225) and PSD categories (β=-0.0627, SE=0.0191, t(16)=-3.2871, p=0.0050).

Supplementary Table 1. Persuasive System Design (PSD) Categories and Features

Principle and definition according to PSD framework		Coded as element included when the web-based intervention:	Example
**Primary Task Support**			
Reduction	A system that reduces complex behavior into simple tasks helps users perform the target behavior, and it may increase the benefit/cost ratio of a behavior.	Specifically divides the target behavior into small, simple steps	An app-based intervention for social anxiety includes a diary for recording daily mood, thereby dividing the target behavior (decreasing anxiety) into small, simple steps of which one is tracking moods
Tunneling	Using the system to guide users through a process or experience provides opportunities to persuade along the way.	Delivers content in a step-by-step format with a predefined order	An app for the prevention of depression that delivers the content in sequential lessons that can only be accessed when the previous lesson is completed
Tailoring	Information provided by the system will be more persuasive if it is tailored to the potential needs, interests, personality, usage context, or other factors relevant to a user group.	Provides content that is adapted to factors relevant to a user group, or when a counselor provides feedback based on information filled out by a participant	An app for treating depression anxiety provides information adapted to patients based on whether they are most concerned about anxiety or depression.
Personalization	A system that offers personalized content or services has a greater capability for persuasion.	Provides content that is adapted to one user (ie, the name of the user is mentioned and/or the user can adapt a part of the intervention)	An app allows users to choose whether they want to see their weekly mood scores on the home page or not
Self-monitoring	A system that keeps track of one's own performance or status supports the user in achieving goals.	Provides the ability to track and view the user's behavior, performance or status	An app allows users to track their symptoms, mood, or behaviors.

Simulation	Systems that provide simulations can persuade by enabling users to observe immediately the link between cause and effect.	Provides the ability to observe the cause-and-effect relationship of relevant behavior	An app includes a before-and-after comparison of a patient who has completed the app treatment.
Rehearsal	A system providing means with which to rehearse a behavior can enable people to change their attitudes or behavior in the real world.	Provides the ability and stimulation to rehearse a behavior or to rehearse the content of the intervention	An app for anxiety starts each lesson with the same meditation.
**Dialogue Support**			
Praise	By offering praise, a system can make users more open to persuasion.	Offers praise to the participant on any occasion	A cognitive behavioral therapy-based app compliments users for completing a homework assignment.
Rewards	Systems that reward target behaviors may have great persuasive powers.	Offers some kind of reward when the participant performs a target behavior relating to the use or goal of the intervention	A web-based intervention for the treatment of social anxiety gives points to participants when they engage in exposure exercises
Reminders	If a system reminds users of their target behavior, the users will more likely achieve their goals.	Provides reminders about the use of the intervention or the performance of target behavior	An app sends an automatic push notifications to remind the participant to log their mood that day.
Suggestion	Systems offering fitting suggestions will have greater persuasive powers.	Provides a suggestion to help the participants reach the target behavior	An app suggests exercises to improve the user’s mood.
Similarity	People are more readily persuaded through systems that remind them of themselves in some meaningful way.	Is designed to look familiar and designed especially for the participant	An app for adolescents with anxiety explains the exercises through a teenager with anxiety.

Liking	A system that is visually attractive for its users is likely to be more persuasive.	Is visually designed to be attractive to the participants	During the design of an app for patients with depression, a representative group is asked for feedback on the design and their feedback is subsequently incorporated in the new design
Social role	If a system adopts a social role, users will more likely use it for persuasive purposes.	Acts as if it has a social role (eg, a coach, instructor, or buddy)	An app incorporates an avatar to guide the participant through the intervention
**Social Support**			
Social learning	A person will be more motivated to perform a target behavior if (s)he can use a system to observe others performing the behavior.	Provides the opportunity and stimulates participants to see others using the intervention or performing the target behavior	An app for depression provides the option, and stresses the importance, of posting cognitive restructuring exercises on the discussion board and commenting on the exercises of others.
Social comparison	System users will have a greater motivation to perform the target behavior if they can compare their performance with the performance of others.	Provides the opportunity for participants to compare their behavior to the target behavior of other participants and stimulates them to do this	An app automatically compares the response of the participant to other users of the intervention
Normative influence	A system can leverage normative influence or peer pressure to increase the likelihood that a person will adopt a target behavior.	Provides normative information on the target behavior or the usage of the intervention	A meditation app for patients with anxiety provides feedback on the amount of meditation the participant completed by comparing it to the meditations completed by well-managed anxiety patients

Social facilitation	System users are more likely to perform target behavior if they discern via the system that others are performing the behavior along with them.	Provides the opportunity to see whether there are other participants using the intervention	An app for depression includes a discussion board for users of the intervention
Cooperation	A system can motivate users to adopt a target attitude or behavior by leveraging human beings' natural drive to cooperate.	Stimulates participants to cooperate to achieve a target behavior	A cognitive behavioral therapy-based app stimulates participants to form groups and to achieve the group goal of a certain number of homework assignments each week.
Competition	A system can motivate users to adopt a target attitude or behavior by leveraging human beings' natural drive to compete.	Stimulates participants to compete with each other to achieve a target behavior	A meditation app includes a leaderboard in which the users who meditate on the most days earn the highest place
Recognition	By offering public recognition for an individual or group, a system can increase the likelihood that a person/group will adopt a target behavior.	Prominently shows (former) participants who adopted the target behavior	An app that treats anxiety includes a testimonial page where successful users of the intervention tell their story
**Credibility Support**			
Trustworthiness	A system that is viewed as trustworthy will have increased powers of persuasion.	System should provide information that is truthful, fair and unbiased.	An app website or App Store profile provides information about the app rather than simply providing biased advertising or marketing information.
Expertise	A system that is viewed as incorporating expertise will have increased powers of persuasion.	System should provide information showing knowledge, experience, and competence.	An app provides information about their core knowledge base and is updated regularly, with no incomplete or out-of-date information.

Surface credibility	People make initial assessments of the system credibility based on a firsthand inspection.	System should have a competent look and feel.	There are only a limited number of, and a logical reason for, ads on a mobile application.
Real-world feel	A system that highlights people or organization behind its content or services will have more credibility	System should provide information of the organization and/or actual people behind its content and services.	An app provides possibilities to contact specific people through sending feedback or asking questions.
Authority	A system that leverages roles of authority will have enhanced powers of persuasion.	System should refer to people in the role of authority.	App quotes an authority, such as a statement by government health office or expert in the field.
Third-party endorsements	Third-party endorsements, especially from well-known and respected sources, boost perceptions on system credibility.	System should provide endorsements from respected sources.	An app shows a logo of a certificate that assures that they use secure connections. An app refers to an award it received for high usability.
Verifiability	Credibility perceptions will be enhanced if a system makes it easy to verify the accuracy of site content via outside sources.	System should provide means to verify the accuracy of site content via outside sources.	Information provided in the app is supported by offering links to other resources such as websites or research articles.

Supplement Table 2. Behavioral Economics (BE) Categories and Features

**Principle and definition according to BE**		**Description in the context of app-based interventions**	**Example**
Loss Aversion	A person is more motivated to prevent a loss than to secure a gain of the same amount. Uses prospect theory[68]	Participant receives something at the beginning of a time period and is encouraged to engage in behaviors to prevent losing that entity	At the beginning of the study, the participant receives 100 points and loses 10 points for each day they do not track their mood
Fresh Start Effect	Temporal landmarks motivate aspirational behavior[11]	Temporal landmarks are demarcated by the app (e.g. start of the week, month, or year), and progress is tracked only in the temporal periods demarcated by the landmarks	Every month, the participant's points or levels reset
Pre-Commitment Pledge	Setting a goal beforehand has been shown to motivate behavior change[12]	Asks the participant to set a goal before starting the intervention	The participant pledges to achieve 1 exposure/week during the intervention before starting the intervention
Lottery	A person is selected at random to receive a prize as a way to motivate the target behavior[13]	Has at least one lottery for which one participant is selected at random to receive a prize	Every participant that tracks their mood at least once during the week is entered into a lottery at the end of the week, and the winner receives $10

Supplementary Table 3. Control Conditions for RCTs

First Author	Publication Year	Name of App	Control Arm
Ben-Zeev[22]	2019	FOCUS	Clinic-based group intervention
Dahne 1[27]	2019	Aptivate	iCouch CBT app; TAU
Dahne 2[26]	2019	Moodivate	MoodKit app; TAU
Enock[28]	2014	Cognitive bias modification of attention (CBM-A)	Placebo CBM-A app; Waitlist
Hur[29]	2018	Todac Todac	Daily mood chart app
Ludtke[31]	2018	Be Good to Yourself	Waitlist
Lukas[32]	2019	MTPhoenix	Waitlist
Ly[33]	2014	Behavioral Activation (BA) intervention	Mindfulness app
Moberg[35]	2019	Pacifica	Waitlist
Mohr[36]	2019	IntelliCare	Coach, with or without recommendations
Roepke A1[39]	2015	CBT-PPT SuperBetter	Waitlist
Roepke A2[39]	2015	General SuperBetter	Waitlist
Stiles-Shields A1[40]	2019	Boost Me	Waitlist
Stiles-Shields A2[40]	2019	Thought Challenger	Waitlist
Stolz[41]	2018	iCBT	PC iCBT; Waitlist

Supplementary Table 4. Treatment of Missing Data in Efficacy Analyses

**First Author**	**How efficacy was computed in the case of drop-out**
Anguera[20]	N/A; Clinical outcomes not reported
Bakker[21]	Removal of missing cases
Ben-Zeev[22]	ITT analyses using mixed-effects models
Bustillos[23]	Removal of missing cases
Caplan[24]	N/A; Clinical outcomes not reported
Chen[25]	Removal of missing cases
Dahne 1[27]	Participants with at least one follow-up assessment were included in generalized estimating equations
Dahne 2[26]	Participants with at least one follow-up assessment were included in generalized estimating equations
Enock[28]	ITT analyses with last observation carried forward
Hur[29]	Removal of missing cases
Inkster[30]	N/A; Only app users who completed both assessments were included in sample
Lim[44]	Removal of missing cases
Ludtke[31]	ITT analyses using multiple imputation (method of imputation was “fully conditional specification,” an iterative Markov chain Monte Carlo method, set as default by SPSS version 24)
Lukas[32]	ITT analyses using multiple imputation (method of imputation was the Markov Chain Monte Carlo multivariate imputation algorithm with ten estimations per missing value)
Ly[33]	ITT analyses using mixed effects models. All analyses used maximum likelihood estimation.
Mehrotra[34]	Removal of missing cases
Moberg[35]	ITT analysis using multilevel modeling
Mohr[36]	Modified ITT analysis with unadjusted outcomes using generalized linear mixed models, assuming a heterogeneous unstructured covariance structure by randomization strata. Secondary analysis used the expectation-maximization algorithm to impute 5 distinct datasets, in which 4-week outcomes were imputed for any participant who did not have at least one follow-up assessment, allowing all participants to be included in generalized linear mixed models. Parameter estimates and standard errors from each of the 5 models were combined and included in the SAS MIANALYZE procedure to derive valid inferences for the parameters of interest.
Norton[37]	Removal of missing cases
Pratap[38]	Generalized estimating equations that account for within-subject correlations of participant responses
Roepke[39]	ITT analyses estimated with the maximum likelihood method in a hierarchical linear modeling framework

Stiles-Shields[40]	Removal of missing cases
Stolz[41]	ITT analyses. Mixed-effect models using unstructured covariance matrices and restricted maximum likelihood estimation with time-points nested within subjects.<br>Analyses were repeated using multiple imputation and repeated-measures ANOVA with time as a within-group factor and treatment condition as a between-groups factor.
Wahle[42]	Removal of missing cases
Watts[43]	Linear mixed-model repeated measures ANOVA with measurement occasion as a within-group factor and intervention as a between-groups factor. Analyses conducted using the MIXED procedure in SPSS Version 19 with an identity covariance matrix.

Supplementary Table 5. App Content

App	Psycho-education	Cognitive Techniques	Behavioral Techniques	Mindfulness	Relaxation	Mood Expression	Tracking Thoughts	Tracking Moods	Tracking Behavior	Tracking Symptoms	Tracking physio parameters
**Aptivate**[27]	X		**X**					X	X		
**BA Intervention**[33]	X		**X**						X		
**Be Good to Yourself**[31]	X	**X**	**X**	**X**							
**Boost Me**[40]			**X**					X	X		
**Calm**[37]	X			**X**	**X**						
**CBMA**[28]		**X**									
**CBMA Control**[28]		Placebo									
**El Buen Consejo Movil**[24]	**X**	X	X				X	X			
**EVO**[20,38]		Video game									
**FOCUS**[22]	X	X	X		X			X			
**Get Happy Program**[43]	X	X	X								
**Health Tips**[20,38]	X		**X**								
**iCBT**[41]	**X**	**X**	**X**		X		X		X		
**iCouch CBT**[27]		X	X								
**Intellicare**[25,36]	X	X	X	X	X			X	X		X

**Mindfulness App**[33]	X			**X**							
**Mobile Sensing and Support**[42]	X	X	**X**	X	X				X		**X**
**Moodivate**[26]	X		**X**					X	X		
**MoodKit**[26]		**X**	**X**			X	X	X	X	X	
**MoodMission**[21]	X	**X**	**X**				X	X	X	X	
**MT-Phoenix**[32]	X	**X**	X		X						
**Pacifica**[23,35]	X	X	X	X	X		X	X	X		X
**Plus Connect**[44]	X	X	X					X			
**PUSH-D**[34]	**X**	**X**	**X**		X			X	X		
**Superbetter CBT**[39]	X	X	X	X					X		
**Superbetter General**[39]	X	X	X	X					X		
**Thought Challenger**[41]		**X**					X				
**Todac Todac**[29]		**X**									
**Wysa**[30]		**X**	**X**	**X**	X						

*Bolded "X"s indicated an app's primary content.

Supplementary Table 6. App Usability Features

App	Safety features			Privacy features		
	Suicidality assessment	Suicidality resources in app	Safety monitoring by study staff	Privacy policy	Information covered by privacy policy	Other privacy features
**Aptivate**[27]						
**BA Intervention**[33]			X			Encryption
**Be Good to Yourself**[31]						
**Boost Me**[40]	X	X	X			
**Calm**[37]				X*	Type of information collected, Rationale for collecting, Sharing of information, User controls	Password
**CBMA**[28]						
**CBMA Control**[28]						
**El Buen Consejo Movil**[24]			X			Username
**EVO**[20,38]						
**FOCUS**[22]			X			
**Get Happy Program**[43]						
**Health Tips**[20,38]						Encryption
**iCBT**[41]						
**iCouch CBT**[27]	X			X*	Type of information collected, Rationale for collecting, Sharing of information, User controls	
**Intellicare**[25,36]			X			
**Mindfulness App**[33]						
**Mobile Sensing and Support**[42]		X				Encryption
**Moodivate**[26]				X*	User controls	

**MoodKit**[26]				X*	Type of information collected, Rationale for collecting, Sharing of information, User controls	Password
**MoodMission**[21]				X*	Type of information collected, Rationale for collecting, Sharing of information	
**MT-Phoenix**[32]						
**Pacifica**[23,35]		X		X	Type of information collected, Rationale for collecting, Sharing of information, User controls	
**Plus Connect**[44]			X			
**PUSH-D**[34]	X	X				Encryption, Password
**Superbetter CBT**[39]						
**Superbetter General**[39]						
**Thought Challenger**[41]	X	X	X			
**Todac Todac**[29]						
**Wysa**[30]				X	Sharing of information, information type collected, reason for collection, user controls	

*Available on website or app profile, external to research publication.

Supplementary Table 7. App Accessibility

App	Accessibility	Availability	Cost
**Aptivate**[27]	Public	Apple App Store	$4.99
**BA Intervention**[33]	Research only		Free
**Be Good to Yourself**[31]	Unclear		Free
**Boost Me**[40]	Public	Google Play	Free
**Calm**[37]	Public	Apple App Store, Google Play	Unclear
**CBMA**[28]	Research only		Free
**CBMA Control**[28]	Research only		Free
**El Buen Consejo Movil**[24]	Research only		Free
**EVO**[20,38]	Research only		Free
**FOCUS**[22]	Unclear		Free
**Get Happy Program**[43]	Research only		Free
**Health Tips**[20,38]	Research only		Free
**iCBT**[41]	Research only		Free
**iCouch CBT**[27]	Public	Apple App Store	$2.99
**Intellicare**[25,36]	Public	Google Play	Free
**Mindfulness App**[33]	Public	Apple App Store	Unclear
**Mobile Sensing and Support**[42]	Unclear	Google Play	Unclear
**Moodivate**[26]	Public	Apple App Store	$4.99
**MoodKit**[26]	Public	Apple App Store	$4.99
**MoodMission**[21]	Public	Apple App Store, Google Play	Free
**MT-Phoenix**[32]	Unclear	Apple App Store	Free
**Pacifica**[23,35]	Public	Apple App Store, Google Play	Free for basic version
**Plus Connect**[44]	Unclear		Free
**PUSH-D**[34]	Unclear	Google Play	Free

**Superbetter CBT**[39]	Unclear		Unclear
**Superbetter General**[39]	Unclear		Unclear
**Thought Challenger**[41]	Public	Google Play	Free
**Todac Todac**[29]	Unclear		Unclear
**Wysa**[30]	Public	Google Play	Free for basic version

Supplementary Table 8. Behavioral Economics Features

App	Loss Aversion	Fresh Start Effect	Pre-Commitment Pledge	Lottery
Aptivate[27]				
BA Intervention[33]				
Be Good to Yourself[31]				
Boost Me[40]				
Calm[37]				
CBMA[28]				
CBMA Control[28]				
El Buen Consejo Movil[24]				
EVO[20,38]				
FOCUS[22]				
Get Happy Program[43]				
Health Tips[20,38]				
iCBT[41]			X	
iCouch CBT[27]				
Intellicare[25,36]	X			
Mindfulness App[33]				
Mobile Sensing and Support[42]				
Moodivate[26]				
MoodKit[26]				
MoodMission[21]				
MT-Phoenix[32]				
Pacifica[23,35]			X	
Plus Connect[44]				
PUSH-D[34]				
Superbetter CBT[39]			X	
Superbetter General[39]			X	
Thought Challenger[41]				

**Todac Todac**[29]				
**Wysa**[30]				
